# An Injectable Hydrogel Platform for Sustained Delivery of Anti-inflammatory Nanocarriers and Induction of Regulatory T Cells in Atherosclerosis

**DOI:** 10.3389/fbioe.2020.00542

**Published:** 2020-06-05

**Authors:** Sijia Yi, Nicholas B. Karabin, Jennifer Zhu, Sharan Bobbala, Huijue Lyu, Sophia Li, Yugang Liu, Molly Frey, Michael Vincent, Evan A. Scott

**Affiliations:** Department of Biomedical Engineering, Northwestern University, Evanston, IL, United States

**Keywords:** hydrogel, atherosclerosis, sustained delivery, regulatory T cells, immunotherapy, nanoparticle, filament

## Abstract

Chronic unresolved vascular inflammation is a critical factor in the development of atherosclerosis. Cardiovascular immunotherapy has therefore become a recent focus for treatment, with the objective to develop approaches that can suppress excessive inflammatory responses by modulating specific immune cell populations. A benefit of such immunomodulatory strategies is that low dosage stimulation of key immune cell populations, like antigen presenting cells, can subsequently propagate strong proliferation and therapeutic responses from effector cells. We have previously demonstrated that intravenous injections of anti-inflammatory nanocarriers provided atheroprotection that was mediated by regulatory T cells (Tregs) upregulated in lymphoid organs and atherosclerotic lesions. Here, we demonstrate an injectable filamentous hydrogel depot (FM-depot) engineered for low dosage, sustained delivery of anti-inflammatory nanocarriers. The bioactive form of vitamin D (aVD; 1, 25-Dihydroxyvitamin D3), which inhibits pro-inflammatory transcription factor NF-κB via the intracellular nuclear hormone receptor vitamin D receptor (VDR), was stably loaded into poly(ethylene glycol)-block-poly(propylene sulfide) (PEG-*b*-PPS) filomicelles. These aVD-loaded filaments underwent morphological transitions to release monodisperse drug-loaded micelles upon oxidation. This cylinder-to-micelle transition was characterized *in vitro* by cryogenic transmission electron microscopy (CryoTEM) and small angle X-ray scattering (SAXS). Following crosslinking with multi-arm PEG for *in situ* gelation, aVD-loaded FM-depots maintained high levels of Foxp3^+^ Tregs in both lymphoid organs and atherosclerotic lesions for weeks following a single subcutaneous injection into ApoE^−/−^ mice. FM-depots therefore present a customizable delivery platform to both develop and test nanomedicine-based approaches for anti-inflammatory cardiovascular immunotherapy.

## Introduction

Atherosclerosis is a chronic inflammatory disorder and a major source of cardiovascular disease (CVD) that involves the accumulation of fatty deposits and inflammatory cells within the intimal walls of arterial vessels (Virani et al., 2020). Lipid-lowering drugs, such as statins, have been widely used in the treatment of CVD. Statins inhibit HMG-CoA reductase and reduce cholesterol levels risk of coronary heart disease and stroke. However, numerous undesired side effects discourage patients from continuing statin treatment, such as stain-associated muscle symptoms, diabetes mellitus, and central nervous system complaints (Thompson et al., 2016). Many patients with reduced low-density lipoprotein (LDL) cholesterol levels still have a high risk of heart attack and stroke (Hu et al., 2003; Sampson et al., 2012; Fernández-Friera et al., 2017). Furthermore, systemic and plaque inflammation are not resolved by statin treatment (Sansbury and Spite, 2016). Recently, attention has shifted from focusing on lipids toward addressing the immune cell-mediated inflammation contributing to CVD. In particular, an imbalance between effector T cells and regulatory T cells (Tregs) can trigger a cascade of inflammatory responses leading to atherosclerosis progression and plaque vulnerability (Ou et al., 2018). Effector type 1 helper T (Th1) cells have been demonstrated to promote the migration of monocytes and T cells into plaques and activate antigen-presenting cells (APCs) by secreting interferon-γ (IFN-γ) and IL-6 (Dietel et al., 2013; Tabas and Lichtman, 2017). However, as an essential immunoregulatory cell population, Tregs induce and maintain systemic immune homeostasis and tolerance by suppressing diverse immune cells, including effector T cells (Th1 and Th17 cells), monocytes, dendritic cells, and natural killer cells, as well as by secreting anti-inflammatory cytokines (IL-10, TGF-β, and IL-35) (George et al., 2012; Chistiakov et al., 2013; Dietel et al., 2013). In the clinic, decreased numbers and dysfunction of Tregs are suggested to be involved in atherosclerosis pathogenesis as evidenced by low levels of circulating and lesional Tregs in patients with vulnerable plaques and acute coronary syndrome (George et al., 2012; Dietel et al., 2013; Jia et al., 2013). Strategies to controllably elicit Tregs *in vivo* are therefore needed to better investigate, develop, and harness their atheroprotective mechanisms.

As essential mediators of immunity and tolerance, APCs play a pivotal role in the induction of Tregs. A variety of approaches to modulate APCs and increase Tregs have yielded promising results in the treatment of atherosclerosis, such as oral administration of anti-inflammatory immunomodulators (Chistiakov et al., 2013), anti-inflammatory cytokine treatment (Ji et al., 2017), and adoptive transfer of tolerogenic dendritic cells (DCs) (Hermansson et al., 2011). Despite these major advances, the clinical use of immunotherapies faces several challenges in both efficacy and safety due to off-target effects. Biomaterials and nanotechnology have been shown to improve the efficacy and safety of immunomodulatory molecules through controlling the colocalization, biodistribution, and release kinetics of drugs (Kim et al., 2011; Shao et al., 2015; Allen et al., 2016). We have previously developed multiple strategies to inhibit inflammation and the progression of atherosclerosis by nanocarrier-enhanced immunomodulation of APCs (Allen et al., 2019; Yi et al., 2019). The nanocarriers were composed of poly(ethylene glycol)-block-poly(propylene sulfide) (PEG-*b*-PPS) and are both non-inflammatory and non-toxic in non-human primates (Allen et al., 2018), humanized mice (Dowling et al., 2017), or mouse models of atherosclerosis (Yi et al., 2016). By adjusting the hydrophilic PEG fraction (F_PEG_) relative to the hydrophobic PPS blocks, PEG-*b*-PPS can self-assemble into diverse nanostructure morphologies that are highly stable *in vivo* due to their lyotropic mesophases and low critical micelle concentration of ~10^−7^ M (Napoli et al., 2002, 2004). Using vesicular polymersomes, we selectively targeted DCs in atherosclerotic mice for intracellular delivery of 1, 25-Dihydroxyvitamin D3 (aVD) (Yi et al., 2019). 1, 25-Dihydroxyvitamin D3 is the active metabolite of vitamin D and has been shown to induce a tolerogenic DC phenotype via interaction with the vitamin D nuclear receptor (VDR) (Mathieu and Adorini, 2002). With a logP of 7.6, aVD stably partitioned into the hydrophobic domains of PEG-*b*-PPS assemblies. Indeed, weekly intravenous administration of these DC-targeted anti-inflammatory polymersomes induced tolerogenic DCs, promoted the proliferation of Tregs and significantly inhibited atherosclerosis in ApoE^−/−^ mice (Yi et al., 2019). Although effective in mice, weekly intravenous administration is not a clinically practical option for human patients, which would instead be better served by a long-term delivery platform to sustain Treg levels after a single injection.

Recently, we reported an injectable filamentous (FM) PEG-*b*-PPS hydrogel, which can be employed for the sustainable delivery of drug-loaded nanocarriers in response to physiological levels of oxidation (Karabin et al., 2018). We found that subcutaneously injected FM hydrogel depots (FM-depots) can sustainably deliver monodisperse micelles (MC) to APC and lymphoid organs such as spleen and lymph nodes *in vivo* for months. We therefore hypothesized that FM-depots might serve as an excellent platform to maintain therapeutic immunomodulation of chronic inflammatory diseases. Although thoroughly characterized for *in vivo* sustained release of diagnostic micelles, FM-depots have never before been employed for the delivery of micelles transporting a bioactive or therapeutic molecule. Here, we demonstrate a controllable anti-inflammatory FM-depot, which can sustainably release aVD-loaded PEG-*b*-PPS MC for months following subcutaneous (s.c.) injection. In this proof of concept work, we aimed to verify that delivery of the immunomodulator aVD in a controlled spatiotemporal manner using FM-depots exhibits the superior capacity to induce Foxp3^+^ Tregs compared to free aVD after 2 months of treatment. This nanocarrier delivery system may therefore serve as an excellent tool to investigate and optimize strategies employing Tregs for CVD immunotherapy and presents new opportunities for long-term, low dosage anti-inflammatory treatment regiments.

## Materials and Methods

### Materials

All chemical solvents and reagents were purchased from Sigma-Aldrich (St. Louis, MO, USA) unless indicated. All antibodies and reagents used for flow cytometry were purchased from BioLegend (San Diego, CA, USA).

### PEG-*b*-PPS Polymer Synthesis

Poly(ethylene glycol)-block-poly(propylene sulfide) copolymers PEG_45_-*b*-PPS_44_ and vinyl sulfone functionalized PEG_45_-*b*-PPS_44_ (VS-PEG_45_-*b*-PPS_44_) were synthesized as described previously (Karabin et al., 2018). Briefly, benzyl mercaptan initiated the living anionic ring-opening polymerization of propylene sulfide. The thiolate was then end-capped with either monomethoxy poly (ethylene glycol)-mesylate to form PEG_45_-*b*-PPS_44_ or α-tosyl-ω-hydroxyl PEG to form OH-PEG_45_-*b*-PPS_44_. The vinyl sulfone functionalized PEG_45_-*b*-PPS_44_ was obtained from converting the hydroxyl group to a vinyl sulfone group. The obtained polymers (PEG_45_-*b*-PPS_44_ and VS-PEG_45_-*b*-PPS_44_) were purified by precipitation in cold diethyl ether, dried under vacuum and characterized by ^1^H NMR (CDCl_3_) and gel permeation chromatography (GPC) (ThermoFisher Scientific) using Waters Styragel THF columns with refractive index and UV-Vis detectors in a tetrahydrofuran (THF) mobile phase.

### Preparation of Filomicelle (FM) Depots

Filomicelles were self-assembled from PEG_45_-*b*-PPS_44_ and VS- PEG_45_-*b*-PPS_44_ polymers via thin-film rehydration method in PBS as described previously (Karabin et al., 2018). Briefly, the mixture of PEG_45_-*b*-PPS_44_ (40 mg) and VS- PEG_45_-*b*-PPS_44_ (10 mg) polymers were dissolved in 1 ml dichloromethane within 1.8 ml glass vials (ThermoFisher Scientific) and placed under vacuum to remove the solvent. The resulting thin films were hydrated with 493 μl of phosphate-buffered saline (PBS) and gently mixed using a Stuart SB3 rotator for at least 36 h. To prepare FM-depots, eight-arm PEG-thiol (10% w/v in PBS solution, Creative PEGWorks) was added to FM solution corresponding to a 1.1:1 molar ratio of thiol:vinyl sulfone. The obtained mixture was vortexed and a Teflon mold (6 mm) was filled with 55 μl of the mixture. The FM-depots were formed after incubation at 37°C in a humidified environment for 30 min.

### Material Characterization

The morphology of nanostructures was determined using cryogenic transmission electron microscopy (CryoTEM). Prior to plunge-freezing, 200 mesh Cu grids with a lacey carbon membrane (EMS Cat# LC200-CU-100) were glow-discharged in a Pelco easiGlow glow discharger (Ted Pella Inc., Redding, CA, USA) using an atmosphere plasma generated at 15 mA for 15 s with a pressure of 0.24 mbar. This treatment created a negative charge on the carbon membrane, allowing liquid samples to spread evenly over the grid. 4 μL of FM samples (10 mg/ml in PBS) was pipetted onto the grid and blotted for 5 s with a blot offset of +0.5 mm, followed by immediate plunging into liquid ethane within a FEI Vitrobot Mark III plunge freezing instrument (Thermo Fisher Scientific, Waltham, MA, USA). Grids were then transferred to liquid nitrogen for storage. The plunge-frozen grids were kept vitreous at−180°C in a Gatan Cryo Transfer Holder model 626.6 (Gatan Inc., Pleasanton, CA, USA) while viewing in a JEOL JEM1230 LaB6 emission TEM (JEOL USA, Inc., Peabody, MA,) at 100 keV. Image data were collected by a Gatan Orius SC1000 CCD camera Model 831 (Gatan Inc., Pleasanton, CA, USA). The images were processed and analyzed using ImageJ.

Small angle X-ray scattering (SAXS) experiments were completed at the DuPont-Northwestern-Dow Collaborative Access Team (DND-CAT) beamline at Argonne National Laboratory's Advanced Photon Source (Argonne, IL, USA) with 10 keV (wavelength λ = 1.24 Å) collimated X-rays. The q-range calibration was performed using a diffraction standard, silver behenate. All the sample measurements were in the q-range 0.001 to 0.5 Å^−1^. The data reduction procedure including subtraction of solvent buffer scattering to obtain a final scattering curve was made using PRIMUS 2.8.2 software. The filomicelle and micelle samples were confirmed using flexible cylinder and polymer micelle model fits, respectively, using SasView.

### *In vitro* Release Study

To determine the release kinetics of payload from FM-depots, lipophilic dye DiIC18(3) (DiI) (ThermoFisher Scientific) was loaded into the polymer mixture at a final fluorophore concentration of 0.067% w/w. The DiI-loaded polymers were mixed with eight-arm PEG-thiol (10% w/v in PBS solution, Creative PEGWorks) to form a DiI-loaded scaffold in Teflon molds. The DiI-loaded FM-depots were incubated in 1 ml DI water with different concentrations of hydrogen peroxide (0, 1, 100 and 500 mM). At different time points (from 1 h to 30 days), 0.5 ml of supernatant was collected and replaced with 0.5 ml fresh DI water. The amount of payload that had been released was then determined using a fluorescence plate reader (SpectraMax M3, Molecular Devices) at an excitation of 549 nm and an emission of 565 nm.

Zetasizer Nano (Malvern Instruments) equipped with a 4mW He-Ne 633 laser was performed to characterize the size distribution of released nanostructures in the supernatant under different oxidation conditions at different time points. Given that the size of micelles in CryoTEM aligned very well with the number average in DLS (Karabin et al., 2018), the number average was used for the representation of the released nanostructure population. The polydispersity index (PDI) was calculated using a two-parameter fit to the DLS correlation data.

### Animals

The apolipoprotein E-deficient (ApoE^−/−^) female mice with C57BL/6 background were purchased from The Jackson Laboratory at 4–6 weeks old. The mice were fed a high-fat diet (HFD, Harlan Teklad TD.88137, 42% kcal from fat) starting at 7 weeks old for 18 weeks until sacrifice. All mice were housed and maintained in the Center for Comparative Medicine at Northwestern University. All experimental animal procedures were performed according to protocols approved by the Northwestern University Institutional Animal Care and Use Committee (IACUC). For each experiment, mice were allocated randomly to each group.

### Treatment

Seven weeks old female ApoE^−/−^ were fed a high-fat diet (HFD, Harlan Teklad TD.88137, 42% kcal from fat) for 3 months before treatment. To prepare the 1,25-Dihydroxyvitamin D3 (aVD)-loaded FM scaffold, 1,25-Dihydroxyvitamin D3 (0.0067% w/w) (Sigma) was loaded into the polymers (*PEG*_45_-*b*-*PPS*_44_ and 20% VS-PEG_45_-*b*-PPS_44_) to form aVD-loaded FMs. The aVD-loaded FMs in PBS were then quickly vortexed with eight-arm PEG-thiol (10% w/v in PBS solution) before use. After 4 months on a high fat diet, 50 μl of various treatment groups were injected s.c. into the mid-scapular region of ApoE^−/−^ mice every month for 2 months: 1, PBS control; 2, free aVD; 3, aVD-FM-depots. The same amount of aVD (8 μg/kg/month) was used in groups 2 and 3. Mice were kept maintaining on a high-fat diet, and their activities were monitored during treatment.

### Flow Cytometry Analysis

After 2 months of treatment, spleen and lymph nodes (two brachial and two axillary from both sides of the mouse) were collected from all groups. Single-cell suspensions from spleen and LNs were prepared as described previously. RBC lysis buffer was used to eliminate red blood cells in spleen samples. Anti-mouse CD16/CD32 was used to block FcRs and Zombie Aqua fixable viability dye was used to determine live/dead cells. For flow cytometric analysis, cells were stained using cocktails of fluorophore-conjugated anti-mouse antibodies: BUV396 anti-CD45, FTIC anti-CD3, PerCP/Cy5.5 anti-CD4, PE anti-CD25, and Alex Fluor 647 anti-Foxp3. After washes, cells were suspended in cell staining buffer and then fixed by IC cell fixation buffer. Intracellular staining of Foxp3 was performed using Foxp3 Fix/Perm Buffer Set following the instruction (Biolegend). At least 200,000 events were recorded per tube on a BD LSRFortessa 6-Laser flow cytometer (BD Biosciences) and data were analyzed with FlowJo software.

### Immunohistochemistry

For immunohistochemical analysis, mice were anesthetized and aortas were carefully harvested after perfusion with PBS under a microscope. The heart with aorta was fixed with 4% paraformaldehyde (PFA)/5% sucrose in PBS solution 12 h at 4°C. The tissue samples were immersed in 15% sucrose solution for 12 h and then 30% sucrose solution for 24 h. The resulting specimens were embedded in Tissue-Tek OCT and frozen at −80°C and then sectioned with a cryostat as described previously (Yi et al., 2019). Briefly, serial sections (10 μm thick) of the aortic roots were collected (10–12 sections per mouse) starting at the appearance of aortic valves. The distance between each section was 150 μm, and totally of 100–120 serial cross-sections were obtained. To determine the immune cell populations in aortic lesions, the slides with multiple frozen aortic root sections were fixed in acetone and washed twice with PBS. Antibodies were performed on consecutive cross-sections for Treg cells (anti-Foxp3, 1:500, Abcam). Slides were stained using the Tyramide Signal Amplification kits in MHPL core facility of Northwestern University. All slides containing the cross-sections were digitally imaged with Leica DM6B widefield fluorescent microscope. An in-house software written in Python was used for automated and quantitative image analysis (Yi et al., 2019).

### Statistical Analysis

The sample sizes were determined based on the results of pilot experiments so that relevant statistical tests would reveal significant differences. For animal studies, 5–8 mice per group were selected in each experiment. GraphPad Prism software (version 8) was used for data analysis. Data are presented as means ± SD. The two-tailed unpaired *t*-test was performed to determine statistical significance.

## Results and Discussion

### Preparation and Characterization of aVD-FM Hydrogel

**Figure 1:**
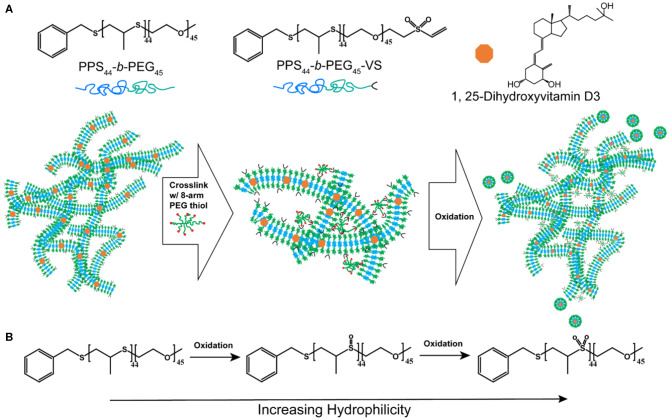
The strategy for sustainably delivering aVD-loaded nanostructures from FM-depots. **(A)** Schematic of the PEG-*b*-PPS copolymers and 1,25-Dihyroxyvitamin D3 (aVD) structures and aVD-loaded filomicelle hydrogel formation. aVD was loaded in filomicelles assembled from PEG-*b*-PPS polymers with 20% vinyl sulfone (VS) functionalization. aVD-loaded FM-depots were formed following crosslinking of VS moieties with 8-arm PEG thiol. aVD-loaded micelles (MC) release from aVD-FM-depots in response to physiological oxidation at the site of injection. **(B)** This thermodynamically driven cylinder-to-sphere transition occurs due to modulation of the hydrophilic/hydrophobic ratio within the filomicelles as the PPS hydrophobic block oxidizes into polypropylene sulfoxide and sulfone derivatives.

**Figure 2:**
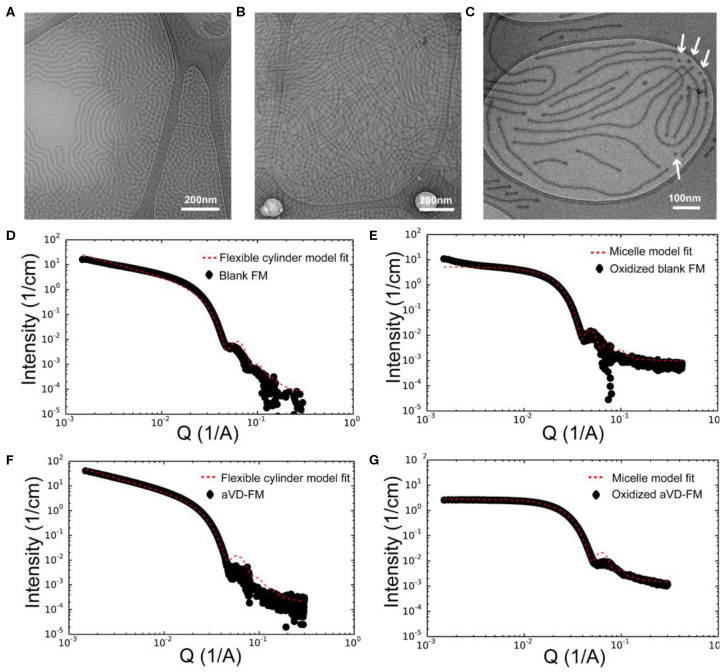
Characterization of aVD-loaded filomicelles. CryoTEM images of filomicelles (FM) **(A)** and aVD-loaded FM **(B,C)**. The white arrows identify the morphological transition from filaments to spherical micelles. Small angle X-ray scattering (SAXS) results are shown for blank FM **(D,E)** and aVD-loaded FM **(F,G)** in PBS solution **(D,F)** or 500 mM H_2_O_2_ oxidizer **(E,G)**.

To achieve sustainable delivery of micellar nanocarriers *in vivo*, an injectable filamentous hydrogel drug depot has been applied. FM-depots allow the sustained delivery of nanocarriers to APC via the cylinder-to-sphere transition at the injection site, wherein the synthetic FM that comprise the depot reassemble into nanocarrier vehicles. In contrast to alternative sustained delivery platforms that employ more stable porous scaffolds to retain nanocarriers for diffusion-based release, FM-depots efficiently degrade into monodisperse nanocarriers to both minimize the amount of administered polymer and avoid chronic inflammation (Yi et al., 2016; Karabin et al., 2018). Cylindrical filomicelles composed of PEG_45_-*b*-PPS_44_ (F_PEG_ ~0.38) and 20% (w/w%) vinyl sulfone (VS)-functionalized PEG_45_-*b*-PPS_44_ were assembled and loaded with aVD using the thin-film hydration method (Figure 1A), as previously described (Yi et al., 2016; Karabin et al., 2018). The crosslinking density can significantly affect the hydrogel stability, degradation, and the rate of nanostructure release. We have previously investigated the physicochemical properties of the crosslinked hydrogels composed of 10, 20, and 30% (w/w%) VS-PEG_45_-*b*-PPS_44._ Mixed filomicelles containing 20% (w/w%) VS-PEG_45_-*b*-PPS_44_ were found to achieve optimal hydrogel stability and degradation rate while releasing monodisperse nanocarriers following crosslinking with 8-arm PEG-thiol. We, therefore, used 20% VS-PEG_45_-*b*-PPS_44_ to form the FM-depots in this study. Given the filamentous structure is critical to the hydrogel formation, the structure of aVD-loaded filomicelles (aVD-FM) was verified using both cryogenic transmission electron microscopy (CryoTEM) and small angle x-ray scattering (SAXS). Furthermore, CryoTEM confirmed that loading of hydrophobic immunomodulator aVD into filomicelles does not change the filamentous structure (Figures 2A,B). aVD-FM showed micron-scale length, which was comparable with the unloaded filomicelles. SAXS scattering profiles of both unloaded and aVD-FM were fitted using a flexible cylinder model with a cylinder length of 2.1 μm and a core radius of 16 nm (**Figures 2D,F**). Suspensions of filomicelles were simply mixed with the 8-arm PEG thiol, which can be spontaneously crosslinked with VS-functionalized filomicelles. The aVD-FM-depot stably formed within minutes. Due to the oxidation sensitivity of PPS blocks, photo- or physiological oxidation converts the hydrophobic poly(propylene sulfide) into more hydrophilic poly(propylene sulfoxide) and ultimately poly(propylene sulfone) derivatives (Figure 1B). Changing the hydrophilic/hydrophobic ratio can trigger a cylinder-to-sphere transition in filomicelles, which we have previously demonstrated via thermodynamic modeling and interfacial measurements to be driven by interfacial tension (Karabin et al., 2018). This morphological transition at the end of the PEG-*b*-PPS filaments into spherical MC, highlighted by white arrows, was clearly observed for aVD-FM using CryoTEM (Figure 2C). Moreover, the SAXS analysis showed that the scattering of the oxidized aVD-FM in 500 mM H_2_O_2_ was fitted well to a MC model with a diameter of 18.4 nm, which is consistent with the oxidized filomicelles without aVD (Figures 2E,G). These data suggested that the aVD-FM demonstrated a similar morphological transition with blank FMs upon oxidation.

### Sustained Release of Drug-Loaded Micelles From the FM-Depots Upon Oxidation

**Figure 3:**
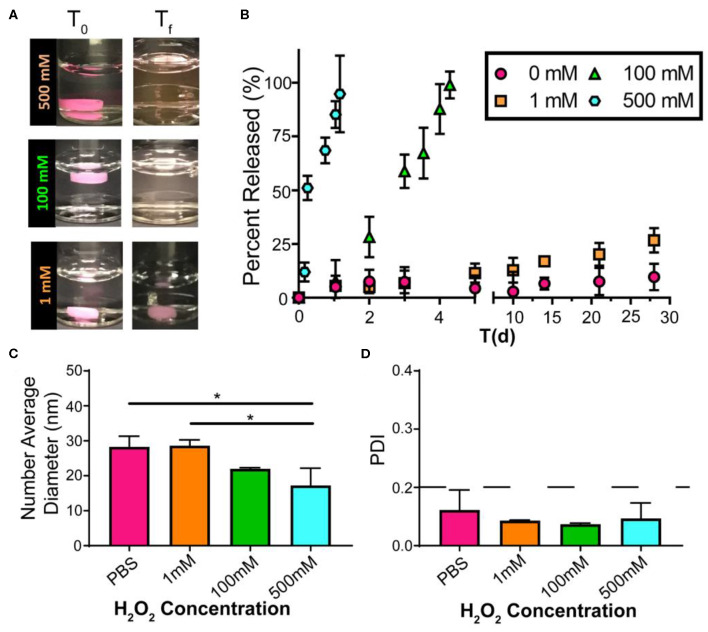
Drug release from FM-depots dependent on the concentration of the oxidizer. **(A)** Pictures of DiI-loaded FM-depots in PBS before and after adding different concentrations of H_2_O_2_ (1, 100, and 500 mM). **(B)** *in vitro* release kinetics of DiI-loaded MC from the hydrogels upon oxidation with different H_2_O_2_ concentrations over 30 days. **(C,D)** Average diameters and PDI of DiI-loaded nanostructures in the supernatant released from DiI-loaded FM-depots. *N* = 3. Two-tailed *t*-tests were used for statistical significance: *p < 0.05.

Excessive ROS is widely acknowledged in atherosclerosis, and high levels of ROS induce systemic oxidative stress, leading to cell apoptosis and redox-dependent signaling disruption (Goncharov et al., 2015). Given the continuous and dynamic production of ROS in the progress of atherosclerosis, it is difficult to accurately quantify the dynamic ROS concentrations in mice. We have previously demonstrated that PEG-*b*-PPS nanostructure morphology is sensitive to physiologic levels of ROS (Du et al., 2017; Karabin et al., 2018), and here we further verified the oxidative responses of FM-depots under various degrees of oxidation *in vitro*. To investigate the release of drug-loaded MC from FM-depots, we chose lipophilic indocarbocyanine dye DiI as a model for hydrophobic drugs. The DiI-loaded FM-depots were immersed in PBS solution with a variety of H_2_O_2_ concentrations (0, 1, 100, and 500 mM). The poor water solubility of DiI induces its aggregation and precipitation in aqueous environments, allowing fluorescence in the supernatant to be attributed solely to DiI-loaded MC released from FM-depots in response to oxidation. The degradation rate of the hydrogels was found to be dependent on the degree of oxidation (Figure 3A), as increasing the H_2_O_2_ concentration accelerated the disassembly of FM-depots. The supernatants were collected and the release of DiI-loaded MC was then quantified for up to 30 days using a fluorescence plate reader. The *in vitro* release kinetics suggested that the release rate of DiI-loaded MC was correlated with the concentration of H_2_O_2_ (Figure 3B), with a rapid release of >90% after only 1 day in the highest concentration solution of 500 mM H_2_O_2_. However, the release rate significantly decreased with decreasing H_2_O_2_ levels, and excellent stability was observed for the 0% H_2_O_2_ sample over the course of 30 days. These data are consistent with oxidation induced reassembly of the filomicelle network within the hydrogel into DiI-loaded MC. The DLS data confirmed the morphology transition from FM to MC with an average diameter ranging from 16 to 30 nm (Figure 3C). It is noticeable that smaller MC diameters were observed in higher concentrations of H_2_O_2_ (500 mM) compared to a low H_2_O_2_ (1 mM) or the PBS control (Figure 3C). These data further indicate that the lower interfacial tension of the oxidized PPS drives the disassembly of FM and reassembly into more thermodynamically stable MCs. In addition, more oxidation may lead to a higher hydrophilic/hydrophobic balance and the formation of smaller MC, as the increased steric repulsion within the thicker hydrophilic corona would induce higher MC curvature. It's noteworthy that the released MC are highly uniform with a polydispersity index (PDI) < 0.1 (Figure 3D). These results verify that FM-depots can serve as controllable delivery systems for the sustainable release of drug-loaded MC under a wide range of oxidative conditions.

### aVD-Loaded FM-Depots Elicit Treg Responses in Atherosclerotic Mice

Although typically associated with modulating bone metabolism, aVD also has potent effects on the regulation of immune responses. Of note, many patients with chronic inflammation like CVD have low levels of the precursor to aVD, 25(OH)VD3, suggesting a potential role for vitamin D in inflammation (Kassi et al., 2013; Yin and Agrawal, 2014). However, clinical studies showed that oral supplementation of vitamin D has very limited effects on systemic inflammation, likely due to the low bioavailability and the broad distribution of the vitamin D receptor (VDR) (Anderson et al., 2010; Carvalho and Sposito, 2015). Intravenous administration of vitamin D in the form of calcitriol is associated with extensive side effects (Goodman et al., 1994), most notably hypercalcemia (Andress, 2001). We have previously demonstrated that encapsulation of aVD in PEG-*b*-PPS nanostructures significantly enhances the efficacy of aVD without side effects and induces tolerogenic DCs, Foxp3^+^ Tregs, and anti-inflammatory effects in atherosclerotic mice (Yi et al., 2019). Building upon this work, an injectable hydrogel delivery system may present a more practical option for the administration of aVD-loaded nanocarriers and provide a means for sustained, low-dosage delivery (Li and Mooney, 2016). It is noteworthy that the monodisperse MC released from FM-depots were within the optimal size range for efficient lymphatic drug delivery following subcutaneous injection (Reddy et al., 2006, 2007). As a proof of concept, we loaded aVD at half the dosage employed in our previous work into FM-depots, sustained the delivery over the course of 2 months and assessed the increased levels of Tregs in the lymph nodes and spleen of ApoE^−/−^ mice.

**Figure 4:**
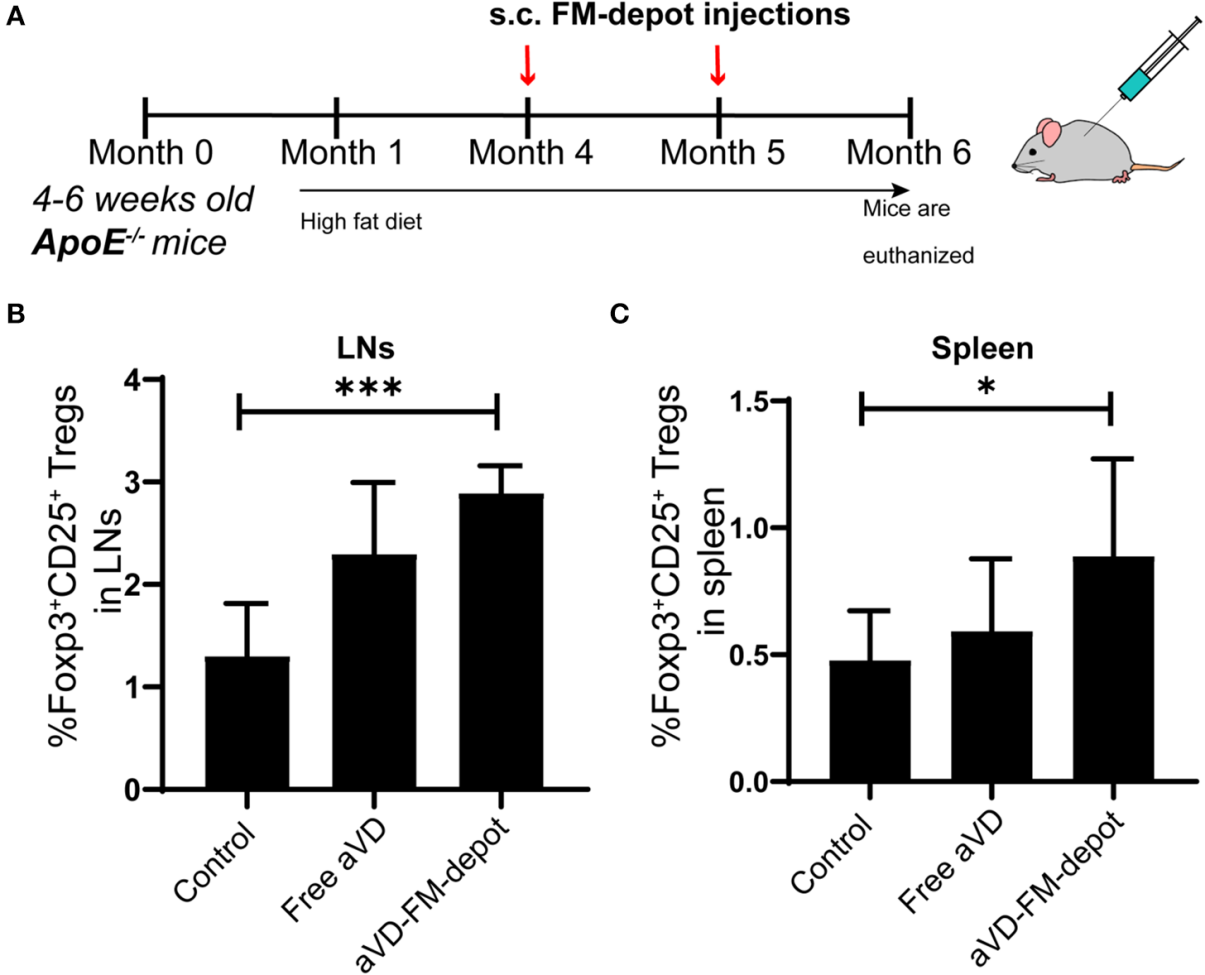
aVD-loaded FM-depots elicit Treg responses in lymph nodes and spleen of ApoE^−/−^ mice. **(A)** Schematic diagram outlining the experimental design for inducing Tregs in ApoE^−/−^ mice. Ten-week old mice were fed with high fat diet for 3 months and then s.c. injected with PBS, free aVD, and aVD-loaded FM-depots monthly. Flow cytometric analysis showed the percentages of Foxp3^+^ Tregs (Foxp3^+^CD25^+^CD4^+^) gated on CD45^+^CD3^+^ live cells in the spleen **(B)** and lymph nodes (LNs) **(C)** of ApoE^−/−^ mice. *N* = 5–8 mice per group. Two-tailed *t*-tests were used for statistical significance: *p < 0.05, ***p < 0.001.

**Figure 5:**
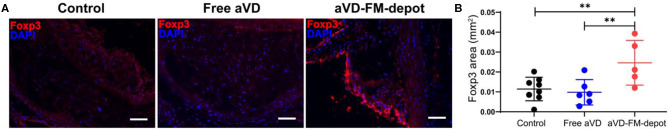
Tregs elicited by aVD-loaded FM-depots migrate to aorta of ApoE^−/−^ mice. **(A)** The expression of Foxp3 (red) was detected by immunofluorescence staining in aortic sinus of ApoE^−/−^ mice. Nuclei were counterstained with DAPI (blue). Scale bar=200 μm. **(B)** Foxp3^+^ Treg content in aorta was quantified in a serial of cross sections using an in-house developed software. *N* = 5–8 mice per group. Two-tailed *t*-tests were used for statistical significance: **p < 0.01.

We evaluated the *in vivo* generation of Tregs by aVD loaded FM-depots in 8–10 weeks old female ApoE^−/−^ mice. After receiving a high-fat diet for 3 months, mice were injected s.c. once per month with a PBS control, free aVD (8 μg/kg/month), or an aVD-loaded FM-depot (8 μg aVD/kg/month) (Figure 4A). After 2 months of administration, the mice were euthanized and no inflammation (swelling or irritation) was observed at the injection site. Activation of antigen-presenting cells (APCs) has been demonstrated to occur within atherosclerotic lesions and in peripheral lymphoid organs, especially draining lymph nodes and spleen, where T cells migrate back to lesions to manipulate local immune responses (Weber et al., 2008; Zhu et al., 2016). The released MC from s.c. FM-depots have been demonstrated to strongly associate with APCs (macrophages and DCs) in both spleen and lymph nodes (Karabin et al., 2018). We hypothesized that our aVD-FM-depots would deliver aVD-loaded MC to APCs and elicit Treg generation in spleen and migration to vascular lesions. We, therefore, characterized Tregs in lymphoid organs of ApoE^−/−^ mice after a 2-month treatment using flow cytometry. The number of Foxp3^+^CD25^+^ Treg cells was significantly increased in CD4^+^ T cell populations in both lymph nodes (*p* < 0.001) (Figure 4B) and spleen (*p* < 0.05) (Figure 4C) for the aVD-FM-depot treatments compared to PBS control. However, no statistical difference was observed in the free aVD group compared to the PBS control group. The T cell responses were further evaluated using immunohistochemistry analysis in serial cross-sections of the aortic root. The levels of Foxp3^+^ Tregs were significantly higher in mice receiving aVD-FM-depots compared to both the free aVD (*p* < 0.01) and PBS controls (*p* < 0.01) (Figure 5). Our results indicated that our FM-depots could sustainably deliver aVD-loaded MC and induce accumulation of Tregs responses for at least 2 months in atherosclerotic mice.

## Conclusions

An injectable s.c. hydrogel delivery system for sustained release of aVD-loaded nanocarriers was characterized and validated *in vivo* to elicit Treg responses in a mouse model of atherosclerosis. As a proof of concept, we used a simple system that delivered solely non-targeted, aVD-loaded MC monthly at low dosages. We verified that aVD could be stably loaded into PEG-*b*-PPS filomicelles without modulating the filamentous structure or inhibiting the crosslinking of filomicelles into hydrogels. Both CryoTEM and SAXS showed these aVD-loaded filaments to undergo cylinder-to-sphere transitions as expected under oxidative conditions to release monodisperse MC. In ApoE^−/−^ mice fed a high-fat diet, significantly increased levels of Tregs were found in the lymph nodes, spleen and atheroma following monthly s.c. injections of aVD-loaded FM-depots. Thus, our work indicated that even at low doses and with less frequent administration, the sustained delivery of aVD via FM-depots could significantly induce the proliferation, expansion and homing of Foxp3^+^ Tregs. This capability may prove to be a promising strategy for clinical translation by offering a practical monthly drug administration regimen and decreased systemic side effects. Although we previously demonstrated that PEG-*b*-PPS filamentous hydrogels could deliver a model fluorescent dye as a payload (Karabin et al., 2018), this is the first demonstration of the sustained delivery of a therapeutic molecule using the cylinder-to-sphere transition to modulate cell function. In future work, we will investigate the therapeutic efficacy of these sustained released FM-depots for inhibition of atherosclerotic plaque development by employing our previously developed nanomedicine-based anti-inflammatory strategies, which include cell-specific targeting (Yi et al., 2019) and alternative atheroprotective immunomodulators (Allen et al., 2019). In summary, this work demonstrates that FM-depots can serve as an effective sustainable delivery platform for the development of combinatorial and sustained delivery approaches to CVD immunotherapy.

## Data Availability Statement

The raw data supporting the conclusions of this article will be made available by the authors, without undue reservation, to any qualified researcher.

## Ethics Statement

The animal study was reviewed and approved by Northwestern University Institutional Animal Care and Use Committee.

## Author Contributions

SY designed experiments, synthesized and characterized the materials and performed the mouse experiments, wrote the manuscript, and discussed the results. NK, SB, SL, and MF synthesized and characterized the materials and discussed the results. JZ, HL, MV, and YL assisted with the animal experiments. ES was responsible for conceptualization, designing experiments, results discussion, and revising the manuscript.

## Conflict of Interest

The authors declare that the research was conducted in the absence of any commercial or financial relationships that could be construed as a potential conflict of interest.
